# Antiviral response within different cell types of the CNS

**DOI:** 10.3389/fimmu.2022.1044721

**Published:** 2022-11-15

**Authors:** Zahra Telikani, Ebony A. Monson, Markus J. Hofer, Karla J. Helbig

**Affiliations:** ^1^ School of Agriculture, Biomedicine and Environment, La Trobe University, Melbourne, VIC, Australia; ^2^ School of Life and Environmental Sciences, Charles Perkins Centre and the Institute for Infectious Diseases, The University of Sydney, Sydney, NSW, Australia

**Keywords:** central nervous system, brain, virus, interferon, innate immunity

## Abstract

The central nervous system (CNS) is a constitutive structure of various cell types conserved by anatomical barriers. Many of the major CNS cell-type populations distributed across the different brain regions are targets for several neurotropic viruses. Numerous studies have demonstrated that viral susceptibility within the CNS is not absolute and initiates a cell-type specific antiviral defence response. Neurons, astrocytes, and microglial cells are among the major resident cell populations within the CNS and are all equipped to sense viral infection and induce a relative antiviral response mostly through type I IFN production, however, not all these cell types adopt a similar antiviral strategy. Rising evidence has suggested a diversity regarding IFN production and responsiveness based on the cell type/sub type, regional distinction and cell`s developmental state which could shape distinct antiviral signatures. Among CNS resident cell types, neurons are of the highest priority to defend against the invading virus due to their poor renewable nature. Therefore, infected and uninfected glial cells tend to play more dominant antiviral roles during a viral infection and have been found to be the major CNS IFN producers. Alternatively, neuronal cells do play an active part during antiviral responses but may adopt differential strategies in addition to induction of a typical type I IFN response, to minimize the chance of cellular damage. Heterogeneity observed in neuronal IFN responsiveness may be partially explained by their altered ISGs and/or lower STATS expression levels, however, further *in vivo* studies are required to fully elucidate the specificity of the acquired antiviral responses by distinct CNS cell types.

## Introduction

The central nervous system (CNS) is an immunologically active tissue, comprising multiple cell populations which belong to different cellular lineages and work in harmony to maintain integrity and function of the CNS (1, 2). Despite its highly protected structure, the CNS can be infected by viruses through several entry portals (3). To protect the CNS, the innate immune system utilizes several types of pattern recognition receptors (PRRs) which are expressed either in the cytosol or on the surface of various cell types, to recognize viral structures and nucleic acids, and induce intracellular signaling pathways, together shaping distinct antiviral responses within alternate cell types (3, 4). Viral sensing by PRRs promotes transcriptional activation of genes which subsequently mediate and define a cell’s antiviral response, including induction of type I interferon (IFN) and the expression of IFN-stimulated genes (ISGs) (reviewed in [4)]. Interactions among the different cell types within the CNS orchestrate innate immune strategies employed to suppress viral infections in the brain (4).

In recent years there has been increasing evidence that region and cell-type specific antiviral responses are induced following viral invasion of the CNS, however, how innate antiviral responses in specific cell types and regions of the brain define host immune protection is not fully understood. In this review, we compare differential intervening factors which are involved in shaping distinct antiviral responses by major cell types of the CNS.

## Cell types of the CNS

**Figure 1:**
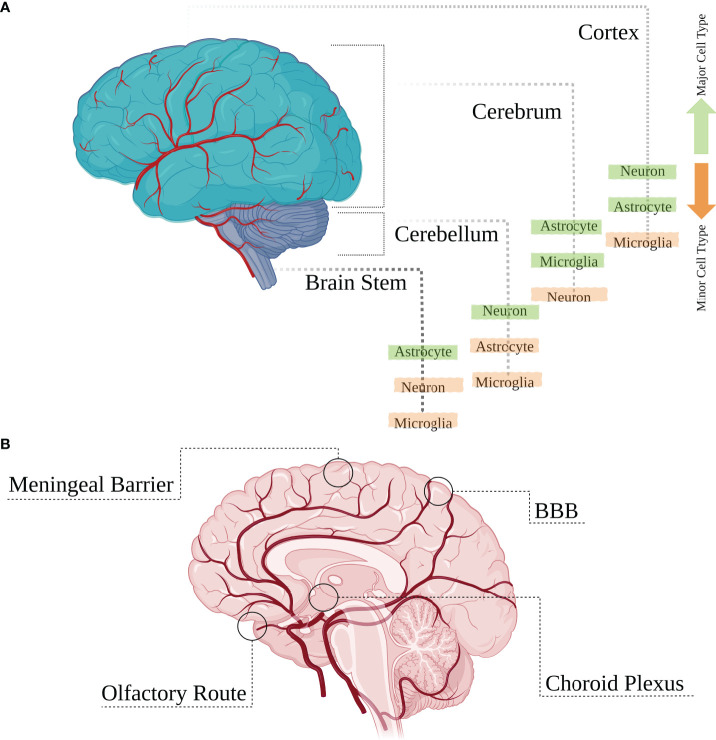
Main brain regions and cell types. **(A)** The brain is mainly composed of the cerebrum, cerebellum and the brain stem which are overlaid by the outermost layer called the cortex. Various cell types are distributed heterogeneously within different regions of the brain with some cell populations having higher or lower densities. **(B)** These brain structures are protected by anatomical restrictive barriers (choroid plexus, meninges, blood brain barrier (BBB), and olfactory epithelium) which create an interface between cerebrospinal fluid (CSF), blood and brain parenchyma and are also considered as entry portals for several viruses.

The central nervous system (CNS) is a constitutive structure of brain and spinal cord. The brain is an assembly of numerous neuronal and glial cell types conserved by restrictive anatomical barriers, including choroid plexus, meninges, the blood brain barrier (BBB), and olfactory epithelium, which are also appropriate niches for viral invasion through providing an interface between cerebrospinal fluid (CSF), blood and brain parenchyma (5). Vertebrate brains generally contain two kinds of tissue: gray matter and white matter. The brain is mainly composed of the cerebrum, cerebellum, and the brainstem (**Figures 1A, B**). In the cerebrum and the cerebellum, white matter is predominantly found in deeper areas with the gray matter coating the white matter; however, grey matter is classed as either superficial or deep and can also be found deep within the cerebrum. The cortex is the outermost layer that overlies most of the other brain structures and is made of gray matter containing many neuronal cell bodies and relatively few myelinated axons, while the white matter harbors neuronal axons. Neuronal cell densities vary across cortical regions which is also true for glial cells; and based on relevant densities data of a mouse brain, the estimated average ratio of cortical neurons to glial cells is about 3:1 (reviewed in [6)]. Astrocytes are more abundant in the white matter and mostly concentrated within the hippocampus and hypothalamus, with microglial cells being more highly distributed in olfactory mesencephalon, basal ganglia and substantia nigra with their lowest density in the cerebellum (7). The cerebellum also includes a high density of neurons, housing half of the total neuron number in the brain. There is also a lower ratio of astrocytes to neurons in the cerebellum than in the cortex or hippocampus (6) (**Figure 1A**).

Many of the major CNS cell-type populations distributed within these brain regions are potential targets for several neurotropic viruses which manage to get past the restrictive barriers mentioned earlier, gaining direct access to these cells. The major CNS cellular population involved in viral infectious challenges of the brain are glial cells. These cells consist of astrocytes and microglia lineage cells, and out-number neurons by some margin, making up around half the volume of the brain [reviewed in (8)]. Astrocytes represent the most abundant fraction of the glial cell populations in the adult CNS and together with microglial cells play key roles in antiviral immune processes shaped against the viral infection (9). Neurons, another major CNS cell-type, are also contributors to CNS antiviral responses [reviewed in (4)], and the outcome of viral infection in each specific cell is modified by distinct antiviral strategies that are orchestrated by various contributing factors that play a role during a cell’s antiviral challenge. Based on several *in vitro* studies of primary cell cultures, most resident cell types within the CNS have the required host cell factors to sense viral infection and trigger multiple innate immune signaling pathways with the purpose of viral elimination (including the production of IFN); however, not many *in vivo* studies have been performed to identify IFN-producing cells within the CNS (10–15). The extensive cellular heterogeneity in the CNS entails vast heterogeneity in cell type-specific immunological responses, however, how this is linked to cell-type specific location and the relative differential transcriptome expression of these cells within the brain, in addition to the other contributing factors needs further exploitation.

### Viral infections of differing brain cell types

Members of several virus families including DNA viruses, retroviruses and RNA viruses have been demonstrated to infect cells in the CNS (**Table 1**). However, not all CNS cell types are equally susceptible or likely to become infected by individual viruses, despite the type of virus, mechanism of survival and route of entry [reviewed in (28)]. Several studies have demonstrated that viral susceptibility within the CNS is not absolute and can differ based on the cell types (e.g., neurons, astrocytes, microglia), cellular subtypes and sub-classes, a cell’s maturation state, or the differential regional location of a cell. These factors have all been shown to play a significant role in the susceptibility of a particular cell to become virally infected, and can influence the outcome of viral infection, although the mechanisms at play are not fully understood.

**Table 1:** CNS cell types susceptibility against several RNA & DNA viruses.

Cell type	Virus	Cell line	Brain region	Model	method	Susceptibility	Ref
**Astrocyte**	TBEV	GFAP+ astrocytes	Cortex, cerebellum, striatum	rat	*In vitro*	Infected/survived	(16)
	TBEV	HBCAs	Cortex	human	*In vitro*	Infected/survived	(17)
	TBEV	hNPCs derived	–	human	*In vitro*	Infected/survived	(18)
	WNV	Human brain tissue derived GFAP+ astrocytes	Cortical	human	*In vitro*	Infected/survived	(19)
	WNV	Primary human fetal astrocyte (U373)	–	human	Ex vivo	Infected/survived	(20)
	ZIKV	GFAP+/SOX2+ primary astrocytes	Human cortical organotypic brain slices	Human	Ex vivo	Infected/survived	(21)
	ZIKV	Fetal atsrocytes	Cortical	Human	*In vitro*	Infected/survived	(22)
	ZIKV	GFAP+	Hippocampus	Rat	*In vivo*	Low-rate infection	(23)
	RABV	GFAP+	Hippocampus	Rat	*In vivo*	differential susceptibility	(24)
	VSV	Primary GFAP+ astrocytes	Whole brain	Mouse	In situ	Infected/survived	(25)
	TMEV	Neonatal Murine derived	Cerebral hemispheres	Mouse	*In vitro*	High replication rate	(26)
**Neuron**	TBEV	hNPCs derived GABAergic neurons	–	Human	*In vitro*	Infected/cell death	(18)
	WNV	Human brain tissue derived neurons	Cortical	Human	*In vitro*	Infected/cell death	(27)
	WNV	Human cholinergic neuronal LAN-2	–	Human	Ex vivo	Infected/cell death	(20)
	ZIKV	Maturing & newborn SATB2+ neurons	Human cortical organotypic brain slices	Human	Ex vivo	Low-rate infection	(21)
	ZIKV	Fetal neurons	Cortical	Human	*In vitro*	Low-rate infection	(22)
	ZIKV	Neun+	Hippocampus	Rat	*In vivo*	Highly Infected	(23)
	RABV	MAP2+ Neurons	Hippocampus	Rat	*In vivo*	Higher susceptibility	(24)
**Microglia**	WNV	Human brain tissue derived CD68+ microglia	Human cortical organotypic brain slices	human	*In vitro*	Low-rate infection	(19)
	ZIKV	IBA1+ microglia	Human cortical organotypic brain slices	human	Ex vivo	Low-rate infection	(21)
	ZIKV	IBA1+ microglia	Hippocampus	Rat	*In vivo*	Low-rate infection	(23)
	VSV	Primary microglia	Whole brain	mouse	In situ *In vitro*	Infected/survived	(25)
	TMEM	Neonatal murine derived	Cerebral hemispheres	Mouse	*In vitro*	Low replication rate	(26)

#### RNA viruses

Examples of cell type specificity in regard to viral infection is well observed following infection of the CNS by members of the *Flaviviridae* family of viruses. Glial cells, in particular astrocytes, have been shown to be highly resilient to Tick-borne encephalitis virus (TBEV) infection, with no alteration in viability observed in primary rat astrocytes 14 days post infection (16). This is mirrored in a model of TBEV infection of primary human brain cortical astrocytes, where a small number of infected cells underwent necrotic cell death, however, most cells remained uninfected by TBEV, *in vitro* (17). The astrocyte`s higher resilience to TBEV infection has also been confirmed by another study revealing higher neuronal susceptibility to TBEV at the peak of infection in comparison to glial cells, specifically astrocytes and oligodendrocytes (18). Conversely, infection of neurons by members of the Flaviviridae family show contradictory results but are generally more susceptible to viral infection. For example, West Nile virus (WNV) infection of neurons obtained from human brain tissue and TBEV infection in differentiated neuronal cells from human neural progenitor cells leads to neuronal cell death (18, 27); however human astrocytes were demonstrated to be as equally susceptible as neurons to WNV infection aside from higher replication rates (27). Another study also demonstrated higher WNV replication rate in human fetal astrocytes with no sign of apoptotic cell-death due to viral infection in contrast to a human neuronal cell line, *ex vivo* (20). Furthermore, primary and fetal human astrocytes appear to also be more susceptible to Zika virus (ZIKV) infection *in vitro*, in comparison to neurons and neural progenitor cells, and are able to withstand higher replication rates in the absence of cell death (21, 22, 29). This is in comparison to a murine model of intracranial ZIKV infection which demonstrated more significant infection in neurons than in glial cells (23), perhaps highlighting differences between human and murine models, or indeed between route of infection.

Glial cell types, including astrocytes and microglia are targeted by many RNA viruses for extended periods of replication, however this does not appear to occur equally, and appears to be virus specific. For example, although neurons are readily infected by rabies virus (RABV), non-neuronal cells seem to be infected abortively, with astrocytes being more resistant than microglia (24). Primary astrocytes and microglial cells are also permissive to Indiana vesiculovirus (VSV) with limitations regarding productive replication, confirmed by VSVinfection of glial cells *in situ* as a result of *in vivo* viral administration (25). Additionally, Theiler’s encephalomyelitis virus (TMEV) infected astrocytes showed higher viral RNA content in comparison to microglial cells, along with a shift in microglia from an anti-inflammatory phenotype (M2) during the early phase of infection to a pro-inflammatory (M1) phenotype during later phases of the infection (26). In the developing rat brain, astrocytes and Bergmann glia cells are among the first cells to be infected by lymphocytic choriomeningitis virus (LCMV) and subsequently spread the viral infection to neurons, however, not all neurons are susceptible to LCMV (30). Additionally, microglia and astrocytes are both permissive to LCMV but differ in their chemokine and/or cytokine response to viral infection (30). The diversity in the ability of RNA viruses to successfully infect glial cells is unlikely due to their repertoire of receptors, given that most RNA viruses can to some extent successfully enter most glial cells, but is perhaps indicative of an intricate balance between specific viral evasion mechanisms and available host cell factors for viral replication. indeed, primary murine astrocytes and microglial cells have been shown to have differential basal transcriptomes and although they both upregulated a core set of transcripts involved in pathogen defense following stimulation with type I IFN, microglial cells had a more extensive and diverse response (31).

There is a concept of selective vulnerability when it comes to neuron maturity, even within the same cell-type population. For example, neurons are a primary target for Sindbis virus (SINV), an Alphavirus from the *Togaviridae* family, which causes neuronal cell-death *via* induced apoptosis. However, neuronal maturation provides protection from viral induced apoptosis, and these cells become resistant to SINV infection (32). In agreement with this, differentiated neuronal cells demonstrate greater susceptibility to RABV along with a higher tendency to sustain viral growth in comparison to primary cultured mouse neurons (33). However, neuronal maturation is not always protective against viral infection, with a neuronal cell model of differentiation demonstrating no changes in susceptibility to LCMV (34), and mitotically active neuronal precursors being shown to be selectively targeted by LCMV within infected neonatal rat brains, with delayed neuronal loss (30). The disparity in generating a persistent viral state in neurons by RNA viruses may reflect differences in neuronal subtypes studied or the relative maturation state as mentioned; however, it is clear that this is an area that requires significantly more investigation to fully understand the role that neuronal maturation plays in viral infection.

#### DNA viruses

Most DNA viruses that cause CNS infections manage to remain undetectable to the immune system due to their inherent capacity to remain latent in the nucleus, however, there are some viruses belonging to the *Herpesviridae* family that can cause persistent infection in neuronal and glial cells within the CNS. Brain cultures obtained from mouse barin, selectively enriched in either glia or neurons showed higher number of murine cytomegalovirus (MCMV) infected astrocytes compared to neurons and microglia, this ratio changed over time with higher numbers of neurons showing evidence of infection after 18 hours, *in vitro* and *in vivo*. In the same study, although MCMV showed no absolute brain cell preference, cortical radial glial cells were the most compromised cell types during infection while striatal neurons demonstrated higher incidence of infection compared to glial cells in the same brain region (35).

Human fetal astrocytes are more susceptible to CMV compared to microglia, however, microglial cells still play important antiviral roles in CMV infection of the CNS modulated by chemokine expression from virally infected astrocytes, *in vitro* (27). Additionally, human fetal astrocytes and microglial cells infected with herpes simplex virus-1 (HSV-1) have been reported to display robust viral replication, *in vitro*, but in contrast to microglial cells, astrocytes do not show signs of cytokine or chemokine induction in response to HSV-1 infection (36, 37). HSV-1 infection has also been demonstrated to lead to strong viral replication in murine neurons and astrocytes, but only weakly in microglia, *in vivo* (38). It now seems that the outcome of a viral infection is dependent on various factors such as the cell type or subtype, and the cellular maturation state. The outcome of a viral infection in the CNS can also be dependent on where the cell is situated within the CNS, regardless of virus species or mechanism of action and route of entry, and this is discussed later. However, alternate factors that may determine the fate of viral infection in specific cell types of the CNS is the acquired innate-immune mediated antiviral pathways following recognition of viral components within distinct cells.

## Antiviral responses within different brain cell types

Specific cell types within the brain act distinctively following detection of viral pathogen-associated molecular patterns (PAMPs), triggering several antiviral signaling pathways (**Table 2**). The effectiveness of these responses appears to vary based on the cell type/subtype that is being infected and the location of that cell within the brain region. The heterogenous antiviral responses adopted by specific cell types within the CNS may be due to the strategic positioning of certain cell types throughout different regions of the brain, with the right cell type in the right region being able to restrict viral replication/spread and minimize damage to non-renewable cells such as neurons. To have a better understanding of the extent to which the CNS antiviral responses can vary in association with cell type specificity and regional heterogeneity within the brain, we must take some steps back to the first virus-cell encounter.

**Table 2:** CNS cell type specific antiviral responses.

	Virus	Cell line	Brain region	PRRs	IRFs	Antiviral response	ISGs	Model	Method	References
**Neuron**	RABV	NT2-N	–	TLR3, RIG-I, PKR	IRF1,7	IFN-	IFIT1,2,4, ISG-20, OAS1,3, MXA,	Human	*In vitro*	(39)
	TMEV & LACV	NT2-N	–	–	IRF7	IFN-α/β (3% of neurons)	Mx1	Murine	*In vitro* *In vivo*	(14)
	WNV	Granule cell neuron Cortical neurons	Cerebellum Cerebral cortex	TLR3 TLR3	–	IFN-β Higher IFN-β	Ifi27, Irg1, Viperin (high IFN responsiveness) Ifi27, Irg1, Viperin (low IFN responsiveness)	–	*In vitro* *Ex vivo*	(40)
	Sendai virus	BE(2)-C Neurons	–	TLR3, MDA5 RIG-I	IRF3	IFNβ, NFKB, PI3K	–	Human Murine	*In vitro*	(41)
	MeV		Hippocampus							
	LACV	NeuN+	Whole brain			No LACV infected IFN producing neuron		Murine	*In vivo* *Ex vivo*	(10)
**Astrocyte**	TBEV	Primary astrocytes	Cerebral cortex			Rapid IFN-α/β response	Viperin & TRIM79α	Murine	*In vitro*	(42)
	TMEV & VSV & RABV	GFAP+	Olfactory bulb	TLR3, MDA5 RIG-I		Type I IFN response (Major producer)	–	Murine	*In vivo* *Ex vivo*	(12)
	LACV	GFAP+	Whole brain	–	–	Major IFNβ producer	–	Murine	*In vivo* *Ex vivo*	(10)
	MHV	CD45- GFP+	Whole brain	TLR3, MDA5 RIG-I (lower basal level)	IRF3, IRF7 (lower basal level)	Delayed but strong IFNβ response	Paracrine IFN responder	Murine	*In vivo*	(13)
**Microglia**	LACV	protein F4/80+	Whole brain	–	–	IFNβ response	–	Murine	*In vivo* *Ex vivo*	(10)
	MHV	CD45int CD11b+	Whole brain	TLR3, MDA5 RIG-I	IRF3 IRF7	Rapid IFNβ response	Autocrine IFN responder	Murine	*In vivo*	(13)
	MHV			MDA5		IFN response				(11)
	VSV	CD11b^+^FCRLS^+^			IRF7	Type I IFN response	*Rsad2*, *Cxcl10*, and *Oas1α*	Murine	*In vivo*	(43)

### Cellular expression of pattern recognition receptors in the brain

Virus infection within specific cells of the CNS triggers activation of several families of pattern recognition receptors (PRRs). Viral nucleic acid is detected through a network of PRRs, including Toll-like receptor (TLR), Retinoic acid-inducible gene (RIG)-I-like receptors, and DNA sensors, expressed by a wide variety of cell types within the CNS. Detection of conserved PAMPs by PRRs activates antiviral signaling pathways in the CNS by promoting transcriptional activation of genes that direct cellular immunity against viruses subsequently leading to secretion of antiviral cytokines such as type-I and -III IFNs and expression of antiviral ISGs (4). It is also important to note that many of the PRRs, and the adaptor proteins controlling the upregulation of type I and III IFNs are also ISGs themselves; therefore expression of IFN in the first instance will enhance the ability of an infected cell and its neighbours to also detect viral infection and subsequently express ISGs to initiate an antiviral environment (44).

TLRs are membrane receptors that detect and become activated by the presence of pathogens *via* an extracellular domain giving rise to several signaling pathways downstream. Intracellular TLRs (TLR 3, 7, 8 & 9) mainly recognize viral nucleic acid, including the recognition of viral double-strand RNAs (TLR 3), single-strand RNAs (TLR 7 & 8) and CpG oligodeoxynucleotide (TLR 9)[reviewed in (45)]. TLRs are expressed by most neuronal and glial cell types within the CNS; however, there is controversy regarding the expression of different TLRs by human neurons. Neuronal TLR expression (TLRs 1-10) have been confirmed at both the mRNA and protein level amongst several species including human, mice and rat [reviewed in (46)]. Specifically, the presence of TLR3 has been confirmed in several neuronal cell lines including SHSY5Y; SH-SY5Y, SK-NSH, BE (2)-C) and primary human neuroblastic cells (47–50). Additionally, the transcripts of all TLRs 1-10 have been detected in primary human neurons as well as NT2-N and CHP-212 neuronal cell lines obtained from cortical brain tissue, although, expression levels vary among these cell types (48). Interestingly, in a human postmitotic neuron-derivative cell line, (mature and differentiated NT2-N), only TLRs 1-4 were detectable which may either be due to differences in the cell’s maturation state or the brain region where cells were obtained (39). In this regard, it must be noted that *in vitro* cell lines can be unreliable regarding their ability to retain TLRs through passaging (47), therefore more work is required to understand the full complement of these receptors in neuronal cell types of the brain.

Like neurons, glial cells also exhibit a wide expression of TLRs, however, this can differ depending on the individual cell type. For example, microglial cells appear to express a more complete repertoire of TLRs in comparison to astrocytes (51), with TLRs 1-9 but not TLR 10 being expressed to some degree at a mRNA level in both murine (52) and human microglia (53). Astrocytes on the contrary, have been demonstrated to express TLRs 1-7, 9 and 10 as shown in human primary astrocytes cultures (54). However, there are some contradictory results surrounding TLR expression in astrocytes, which may be accounted for by culture cell differences, or activation/stimulation status in the studies [reviewed in (55)]. For instance, TLR 2 and TLR 3 are the prevalent TLRs in astrocytes and are both highly expressed in RNA and protein levels in human white matter samples and human astrocyte cultures of embryonic origin, respectively (53, 56), however, an alternate study reported TLR 2 mRNA level as negligible in astrocytes obtained from human CNS tissue (cerebral hemispheres) obtained from fetuses (54). Interestingly, TLR 6, 7 and 8 mRNA and protein levels in normal human astrocytes isolated from the cerebrums of human fetuses are either at their minimum or completely absent (57). Members of a set of TIR domain-containing adaptors such as MyD88, TRIF, TIRAP/MAL, or TRAM are recruited differentially by individual TLRs. The myeloid differentiation primary response gene 88 (MyD88) pathway activates nuclear factor κ-light-chain-enhancer of activated B cells (NF-κB), leading to subsequent induction of inflammatory cytokine genes (58). MyD88 is recruited to cell surface TLRs (TLR2 and TLR4) by TIRAP, a sorting adaptor which has also been shown to participate in signaling through endosomal TLRs such as TLR9. Recruiting TIR-domain-containing adapter-inducing interferon-β (TRIF) to TLR3 and TLR4, gives rise to an alternative pathway resulting in the induction of type I IFN and inflammatory cytokine genes through activation of interferon regulatory factor 3 (IRF3), NF-κB, and MAPKs [reviewed in (45, 46)].

Another PRRs subfamily expressed by different cell types within the CNS is retinoic-acid-inducible gene I (RIG-I)-like receptors (RLRs), which are located in the cytoplasm and consist of RIG-I, melanoma differentiation-associated gene 5 (MDA5; also known as IFIH1) and laboratory of genetics and physiology 2 (LGP2) [reviewed in (3)]. RLRs recruit adaptor molecules Mitochondrial antiviral-signaling protein (MAVS) and Tumor-Necrosis Factor (TNF) Receptor Associated Factor (TRAF) which leads to NF-κB and IRF3 activation, followed by production and release of type 1 IFN and proinflammatory cytokines with antiviral properties (4, 59). All three members of RLRs family have been reported to be expressed in resident cells within the CNS, including neurons, astrocytes, and microglia, with astrocytes and microglial cells are considered to be the predominant source of MDA5 and RIG-I. The role for these receptors in the CNS has been mostly studied in microglia, but astrocytes and neurons express functional levels of some of these receptors (3, 60).

DNA viruses are mainly recognized by the cytoplasmic viral DNA sensor, cGAMP synthase (cGAS), which signals through an adaptor molecule, stimulator of IFN genes (STING). STING recruits TBK1, and this results in the activation and nuclear translocation of IRF3 and induction of type I IFNs. Most studies performed in this area are around HSV-1 infection, showing higher viral load and cell death in STING-deficient mice along with a failure in type I IFN production [reviewed in (4)]. It has been shown that cGAS works in a concerted manner with RIG-I in neuronal cell lines, in order to mount an efficient innate immune response and decrease viral load, even against RNA viruses like Japanese encephalitis (JEV) (61). Human microglia and astrocytes appear to express cGAS-STING viral sensing components, however, there are limited studies surrounding the presence of the cGAS-STING pathway within different CNS cell types (62). In general, neurons, astrocytes, and microglia express PRRs and are capable of triggering downstream signaling pathways following viral recognition. However, there are some discrepancies regarding expression level of these PRRs and which ones are activated post infection in each specific cell type within the CNS. Interestingly, active engagement of the epidermal growth factor receptor (EGFR) has also recently been shown to drive alternate antiviral pathways in numerous cell lines, including astrocytes, and is activated by many viruses of the CNS, however, has not been investigated in the context of the CNS, and may be a contributing factor to the diversity seen in antiviral cell type responses of the CNS (63–68).

## Differential type I IFN/IFNAR responses in the CNS

Infection by many viruses is associated with type-I IFN production, particularly in the early stages of infection. However, viral nucleic acid sensing pathways and the main protein players involved can vary among different brain cell types, and therefore may play a role in shaping a cell-type specific IFN response within different brain cell populations (69). It is clear that many CNS resident cell types, including neurons, astrocytes and microglia, are capable of mounting an effective type I IFN response against viral infections; however, there is evidence suggesting these cell types act differentially in response to type I IFN through IFNAR induced signaling pathways (31, 70). For the CNS to defend itself effectively against viral infections, many residential cells utilize intrinsic and extrinsic innate immune responses which appear to be distinct based on cell type/subtype as well as the regional diversity of these cell types.

### Regional diversity

**Figure 2:**
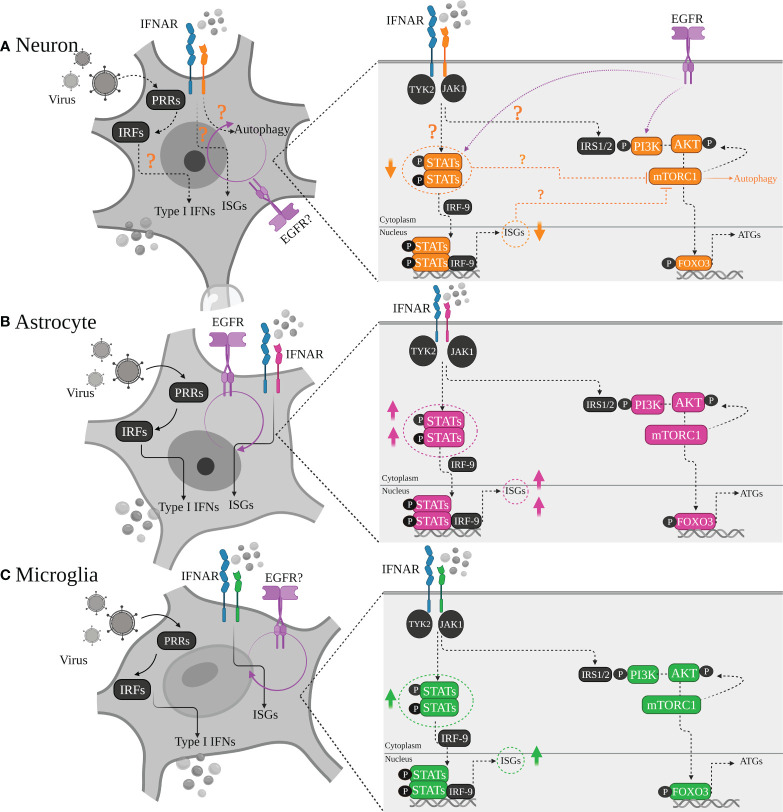
Differential type I IFN/IFNAR responses of major brain cell types. **(A)** Neurons, **(B)** Astrocytes and **(C)** Microglia are all equipped with machinery for viral sensing and can mount an antiviral response, particularly by inducing type I IFN production through various signaling pathways which intersect at activation of different protein kinases and IRF transcription factors, leading to induction of IFN gene transcription downstream. Although all tree cell types produce type I IFN, with astrocytes being major IFN-β producers, and microglia as the main source of IFN-α, there is a heterogenicity regarding neurons IFN responsiveness. Some neurons prefer autophagy rather than ISGs upregulation and induction of a typical antiviral state. This has been linked to lower STATs or basal ISGs levels in neurons compared to other cell types depending on cells developmental state, regional diversity, neuronal sub-types which may play definitive roles in shaping the outcome of the viral infection.

There is increasing evidence that region-specificity of induced type I IFN responses occurs within the brain, as cell types/subtypes of the same cell lineage situated within different brain regions can respond differentially, even with infection of the same virus. This has been linked to differences in the ability to mount a type I IFN response, and localized expression of ISGs (**Figure 2**). For example, murine cortical neurons from the cerebral cortex showed higher permissivity to WNV replication in comparison to granule neurons from the cerebellum, *in vivo* (40). The enhanced antiviral response induced by granule cell neurons was shown to be correlated with the epigenetic state and micro-RNA mediated regulation of ISGs, as these cells showed higher basal and IFN-β treated ISG expression, including viperin expression (a potent antiviral gene), compared to cortical neurons (40). Incapability of granule cell neurons within the cerebellum, to restrict viral infection has also been shown *in vitro* following TBEV and ZIKV infection, compared to primary cortical neurons, due to higher ISG expression levels, including viperin, within neurons of the cortex (71). Interestingly, human and murine cortical astrocytes demonstrated higher basal and

IFN-induced expression levels of ISGs and PRRs than astrocytes of the cerebellar cortex in a murine model of WNV, highlighting the observation that specific cell types may be intrinsically primed for more rapid IFN responses in comparison to neurons (42). Additionally, IFN-β mediated signaling pathways in the hippocampus have been shown to create a refractory state in astrocytes and microglial cells against measles virus (MeV) infection using organotypic brain cultures model, *via* upregulation of ISGs such as MX1 which was not the case for neuronal cell types of the same region (72). These diverse type I IFN response programs within brain cell types may also act in a long-distance manner, through IFN-β signaling pathways and ISG upregulation of regionally distant and/or even uninfected cells. This has been shown to occur in VSV infection of the olfactory bulb, which led to type I IFN induction and upregulation of ISGs in uninfected cells located within the cerebellum (73).

Interestingly, in comparison to other regions of the brain, the cerebral cortex appears to be more vulnerable to viral infections due to its lower ISG expression, regardless of cell type (13, 40). This appears like a rational strategy to adopt, to sacrifice cells that are anatomically closer to restrictive barriers which would be utilized by viruses to invade the CNS. In this manner, the brain could restrict viral infection in such regions and signal other cell types in distant regions for support through enhanced type I IFN responses and upregulation of antiviral ISGs.

### Cell type specificity

**Figure 3:**
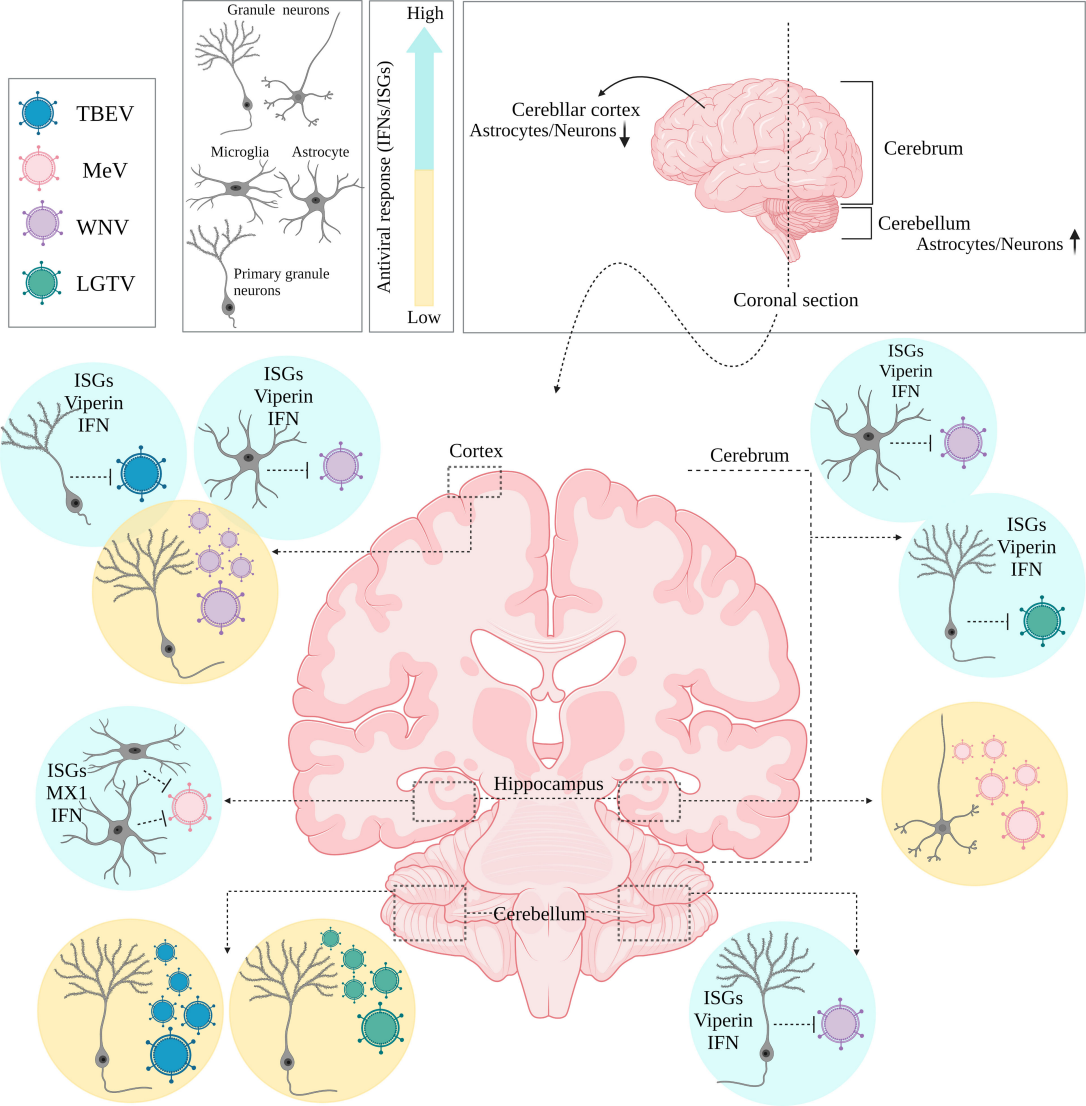
Regional diversity of innate immune responses within the brain. Coronally section of the human brain. Differential permissiveness of various cell types (Granule cell neurons, astrocytes and Microglia) situated in distinct anatomical locations (cortex, cerebrum and cerebellum) within the brain to several viruses; highlighting potent contribution of type I IFN mediated signaling pathways and over expression of relative ISGs.

The diversity of antiviral responses within the CNS could also be looked at from the perspective of cell-types and specific induced signaling pathways by them, including autophagy, cell death pathways and those triggered by interferon. Type I IFN mediates signaling primarily through the Janus kinase (JAK/STAT) pathway (74). IFN binds to its receptor IFNAR1/2, triggering the phosphorylation of STAT1 and STAT2 *via* tyrosine kinase (TYK) 2 and JAK1. The STATs phosphorylation with the association of IRF9 then leads to form a transcriptional activator complex which subsequently is translocated to the nucleus and binds to the promoter regions of IFN-stimulated genes (ISGs), and activates their transcription (74). It is clear that the three major cell types of the brain (Neurons, Astrocytes, Microglia) are intrinsically equipped to sense viral infection, however, their activation of specific downstream signaling pathways following viral detection could differentially contribute to the outcome of the viral challenge (**Figure 3**).

### Astrocytes and microglia

Astrocytes are considered the main IFN-β producers of the brain in many cases of neurotropic viral infections, including RABV, TMEV, and VSV, and have been established to sense viral components in a TLR3/RIG-I/MDA-5 mediated pathway leading to IFN-β induction in theses cell types (10, 12). This is consistent with other results on constitutive TLR3 expression and type I IFN (mainly IFN-β) production in poly (I:C) stimulated human brain astrocytes and neonatal rat astrocyte cultures (13). Detection of TLR3 protein through cell surface and intracellular staining along with activation of IRF3, STAT1 and IκBα by poly (I:C) treatment observed in human brain astrocytes proved to be sufficient for an effective type I IFN response (75). This is also true in the case of flavivirus infection of astrocytes which leads to type I IFN response and upregulation of ISGs like viperin (67, 76). Additionally, Studies using astrocyte cultures revealed that Type I IFN was upregulated in a TLR3-dependent manner in response to viral (murine hepatitis virus [MHV]-A59, TMEV) infection (13, 77).

Microglia, another glial cell type, with astrocytes are considered the main source of IFN-β within the brain in many virus infections (10, 78). Microglial cells, either alone or together with astrocytes, were shown to be the predominant producers of type I IFN upon infection with La Cross virus or coronavirus mouse hepatitis virus; and interestingly this cell type specificity was retained even in the presence of a mutant form of La Cross virus, unable to abrogate host IFN production (10, 11). These cells are critical component of CNS innate defense mechanisms playing major antiviral roles utilizing microglial sensors TLR3 and TLR7 located either in endosomal compartments or on the cell surface, with an involvement of RIG-I-like receptors such as RIG-I and MDA5 to induce an appropriate IFN response. Exposure of primary microglia, mouse and human microglial cell lines to TLR3 ligand poly (I:C) or TMEV showed the expression of several cytokines and chemokine including IFN-β (79).

Unlike neurons, glial cells across different regions of the CNS have been demonstrated to be capable of inducing type I IFN responses against viral infection in a mouse model, and don’t appear to be impacted by subtypes or developmental states (12). However, astrocytes play a more prominent role regarding IFN-β production. Based on several studies, microglial cells are considered to be more productive than responsive, in contrast to astrocytes regarding type I IFNs. Interestingly, upon intranasal VSV instillation, it has been shown that lack of IFNAR signaling in microglial cells does not affect VSV viral spread in the olfactory bulb; however, IFNAR signaling on neurons and astrocytes is essential for the control of viral spread in the CNS *via* the olfactory route (80). Additionally, analysis of gene expression associated with IFN-α/β function post MHV infection indicated poor basal level of ISGs and IFN-β mRNA induction in astrocytes compared to microglia. On the other hand, astrocytes show rapid and higher responsiveness to type I IFN production following MHV viral infection which underpins the role of microglia as a type I IFN source and astrocytes as a responder (13).

### Neurons

Although several studies have presented neurons as being both IFN-α/IFN-β producers and responders, this does not appear to apply to all neuronal cell populations equally following viral infection (10, 14, 39–41). Many studies have exemplified the ability of neurons to mount an effective type I IFN response, including RABV infection of differentiated human neurons, which triggers a typical antiviral immune response (39). The most up-regulated genes in these neurons following RABV infection belong to the immunity cluster, particularly genes under IFN-β control including, IFIT-1, IFIT-2, IFIT-4, ISG-20, GBP-5, GBP-1, OAS-1, OAS-3, and MxA; genes coding for the interferon regulatory factors IRF7 and IRF1; genes coding for the activators of transcription STAT1, STAT2, and NF-κB; and genes which can sense dsRNA, in particular PKR, RIG-I, and TLR3 (39). Granule cell neurons within the cerebellum have also been shown to produce IFN-β post WNV infection and upregulate expression of ISGs like Ifi27, Irg1 and viperin (40). Additionally, neurons from the murine brain infected with either LACVdelNSs or TMEV have also been shown to produce IFN-α and IFN-β *via* upregulation of IRF7, and to subsequently respond to type I IFNs by Mx1 upregulation, however, the ability for neurons to produce type I IFN was only observed in a small proportion of the infected neuronal population (14).

Neuronal maturation state has also been demonstrated to be an important factor in the ability of these cells to respond to viral infection, with neurons from human cerebral organoids showing differential responses to LACV infection based on their maturation state. Although both progenitor and committed neurons were susceptible to LACV, committed neurons underwent apoptosis following infection, which was hypothesized to be due to their lower expression level of ISGs (81). The correlation between neuronal developmental state, specific cell-type ISG levels and type I IFN/IFNAR signaling pathways has also been investigated in another study post infection with alphaviruses (82). In this study, a differentiated neuroblastoma cell line BE (2)-C, showed an enhanced type I IFN response by upregulating STAT-2 and IRF9, and subsequent increases in both the IFNAR2a isoform and the signaling-competent IFNAR2c transmembrane isoform compared to undifferentiated neuronal cells (82). These contradictive results may be due to the different experimental systems, monolayer versus organoids, or differences in the viruses used in these studies. Despite these discrepancies, the role of neurons developmental state in shaping the antiviral response adopted by these cell types is interesting and requires further investigation to fully comprehend the effect this may have on the ability of these cells to respond to viral infection.

Neurons are capable of inducing sufficient innate immune responses following viral infection, however, these responses seem to vary depending on the virus, neuronal cell line and neuronal maturation (10, 14, 39–41). Based on innate immune response strategies in the CNS, putting neurons in danger of altruistic apoptotic cascades and probable irreversible damage following viral infection is not ideal for the CNS. Therefore, it has been hypothesized that non-neuronal CNS resident cells, such as astrocytes and microglia, promote neuronal survival by modulating neuronal PRR responses and controlling viral replication [reviewed in (83)].

Unlike glial cells with their strong ISGs repertoire, some neuronal populations express very low basal level of IFN and ISGs, therefore may differ in their responsiveness to type I IFN. In this regard, some neurons have been shown to either respond poorly to IFN treatment and/or preferentially choose autophagy as their antiviral mode of action. For instance, hippocampal CA1, CA2 and CA3 neurons display differential MX1 expression in response to IFN treatment (84), and dorsal root ganglionic neurons are poorly responsive to type I IFN treatment and are more likely to undergo autophagy to enable herpes virus clearance (85). A possible explanation for discrepancies observed in neuronal cell populations regarding type I IFN responsiveness, may be due to their poor ISGs repertoire compared to glial cells. Data from several studies have established transcriptional upregulation of ISGs may vary from weak to strong depending on specific cell-type and also where it is located within the CNS, for example, IFN treatment of primary neurons infected with TMEV and VSV triggers a surprisingly weak antiviral resistance and is ineffective to reduce viral replication (70). This weak resistance observed in post-mitotic dorsal route ganglion neurons has been suggested to be linked to ISGs lower basal expression levels compared to other mitotic cells like mitotic fibroblasts and selective upregulation of these genes in response to type I IFNs which may in part explain why neurons respond to IFN in a heterogenous manner depending on maturity state and/or brain region (85–87). Since some ISGs, such as STATs, are critical to mediate the IFN response, it has been speculated that STAT1 expression in a proportion of neurons may not reach the threshold of responsiveness post type I IFN treatment. This is while primary hippocampal neurons respond to type I IFN with signature ISG expression (STAT1 & STAT2) compared to primary embryonic fibroblasts (88). This has also been observed in rat hippocampal neurons, which are even less responsive than mouse neurons (89). Thus, signaling pathways involved in ISGs induction may contribute to shaping distinct antiviral responses within different cell-types of the CNS.

### Autophagy, a cell-type specific choice of antiviral response

Almost all CNS resident cell types contribute to type I IFN production following virus invasion; however, not all these cells respond similarly to IFNs which may subsequently define the induced antiviral response of a specific cell type and the outcome of the host-virus survival challenge (**Figure 3**). Both antiviral and cell death pathways are key components of host antiviral defense and can be activated by type I IFN receptor signaling (90). In most tissues, cell death is considered a desired mode of action against viral infection which basically is applied to prevent viral replication and spread [reviewed in (91)]. However, loss of non-renewable cells such as neurons is not affordable by vertebrate hosts (92–94). In parallel, autophagy is an antiviral defense mechanism that does not require cell death for virus control. Autophagy has been shown to play a direct role in control of viral replication by engulfing cytosolic virions for lysosomal degradation (94). As mentioned earlier, autophagy may be considered a favored mode of action adopted by some neuronal cell populations (85, 95), which is not the case in glial cells. For example, in contrast to mitotic cells such as MEFs and mouse keratinocytes, post-mitotic mouse primary neuronal cells predominantly use autophagy over type I IFN as a viral control mechanism; as HSV-1 infected and poly I:C stimulated DRG sensory neurons produce type I IFNs, but to a strikingly lower extent than non-mitotic cells; with autophagy seeming to be required to block HSV-1 infection in these cells (85). Consistent with this observation, HSV-1 infected neurons in an alternate study also failed to induce type I IFNs, however autophagy induction was not investigated; and autophagy induction in neurons is also critical in clearance of Sindbis virus (95, 96). In contrast, HSV-1 infection of astrocytes and microglial cells has been shown to be both IFN productive and responsive, with microglia cells responding to HSV-1 infection with the most potent type I IFN response in a STING-dependent manner, and ISG upregulation being the most highly induced in astrocytes (38). In contrast to neuronal cells, the RNA and DNA virally induced type I IFN response and ISG production seems to be sufficient for mounting an effective viral clearance in glial cells, without the induction of autophagy (42, 43).

The mitotic nature of cells may in part explain why neuronal cells appear to adopt differential antiviral strategies from glial cells; perhaps to minimize cellular damage whilst inhibiting viral spread, and to protect other non-renewable cells. Interestingly, in addition to the JAK/STAT pathway, type I IFN also activates the PI3K/AKT/mTORC1 signaling pathway, which is required for transcription and/or mRNA translation of ISGs (97–99) and promotes cell survival (100, 101). This pathway is also important in regulating autophagy (reviewed in (102)]. A wide range of extracellular signals activate PI3K, for example, PI3K is activated after phosphorylation of insulin receptor substrate 1 (IRS1) [reviewed in (103, 104)]. Interestingly, type I IFN can also induce phosphorylation of IRS1, providing the docking site for PI3K (105), and therefore, type I IFNs have been suggested to take part in autophagy induction *via* a PI3K/AKT/mTORC1 signaling pathway [reviewed in (74)]. In this regard, it has been suggested that type I IFNs may block mTORC1 function and induce autophagy, however the underlying mechanisms are not fully understood, and its potential role in the CNS is also understudied. It is possible that IFN induction of autophagy *via* a PI3K/AKT/mTORC1 may also play a role in the cell-specific antiviral responses induced by distinct cell types within the CNS and differentially activated signaling pathways downstream of the type I IFN receptor.

## Insights from the advent of single cell analysis technologies

Although *in vitro* studies have extended our knowledge surrounding cell type specific responses in cells of the CNS, the very recent advent of single cell transcriptomics should allow a better picture of cell type specific anti-viral responses during *in vivo* infection models. Unfortunately, to date, this technology has not been used to its full potential for *in vivo* studies of virus infection in the brain. This is mainly due to the challenging nature of separating out individual cell types, where they have been found to be lost in the process of dissociation from intact tissue into single cell suspensions [reviewed in (106)].

Despite the challenges associated with single cell transcriptomics in the brain, great efforts have been made in resolving regional cell type landscapes of mainly homogeneous cell populations within the CNS during virus infection. For example, transcriptional changes in the mouse dorsal raphe nucleus following intracranial injection of RABV revealed that the differential cell types varied in their type I and II IFN responses, with these responses being mainly mediated by a small subset of microglial cells (107). Additionally, single-cell transcriptomic analysis has revealed a microglial-like cell subset which appear to be a target for HSV-1 infection in murine brains and display a distinct inflammatory signature with an impact on disease phenotype (108). Single cell technologies have also been utilized to study the transcriptional changes in different brain cells of SARS-CoV-2 patients; and although the virus itself wasn’t present in these patient brain samples, this technology was able to demonstrate a clear change in cellular transcriptional profiles within microglial cells, astrocytes, oligodendrocytes, and excitatory neurons residing in the pre-frontal and frontal cortex, and the choroid plex (109, 110); Interestingly the use of this technology was also able to demonstrate that microglial cells in particular appear to display a persistently activated innate immune state, providing us further insight into the cell type specific immune response to SARS-CoV-2 systemic infection (110).

Given there are only a handful of studies to look at the single cell transcriptome of the brain tissues following infection, and these studies have not yet looked at the full cellular landscape of the brain during a viral infection event, there is still much to learn. Lessons could be taken from alternate fields, where cellular landscapes have been sourced from individual regions of the brain to address the gap in our knowledge surrounding which cell types/cell subsets are the main drivers of immune responses within the CNS *in vivo*; and how this may differ regionally.

## Conclusions and future perspectives

Most cell types of the CNS are equipped to sense viral infection and take an active and unique role in antiviral strategies that protect themselves, as well as bystander cells by being both type I IFN responsive and productive. However, not all cell types are equally susceptible to viral infection, with differential CNS cell types adopting distinct antiviral strategies to defend against viral infection within its own microenvironment, which defines the outcome of the viral infection. In this context, although glial cells are major type I IFN producers and responders due to their stronger expression of STAT and ISG repertoires, astrocytes tend to adopt a more responsive role with microglial cells being more productive, and potentially acting as an alarming signal for adaptive immunity, driving major anti-inflammatory roles during viral infection (111). In contrast, neurons may respond differentially to type I IFN based on their maturity state or brain region they are located in, which may define their ISG repertoire and the relative alterations in ISGs expression in response to IFNs; with further work in the field required to fully understand the role of autophagy induction in preference to ISG upregulation to inhibit viral spread and minimize further damage to non-renewable neurons.

Our understanding of the role of individual cells of the CNS in mounting a protective antiviral response is still in its infancy, with many conclusions derived from studies only involving individual cell types from variable origins in either an *in vitro* or *ex vivo* manner. The advent of improved single cell analysis, and the continued advancement in the space of single cell proteomics, metabolomics and lipidomics is likely to reveal novel insights into how the cells of the CNS act collaboratively during *in vivo* viral infections, providing an improved knowledge of intercellular communication towards design of novel therapeutics to combat viral infection of the CNS.

## Author contributions

ZT wrote the manuscript. All remaining authors researched data for the article, made substantial contributions to the discussion of the content and reviewed and/or edited the manuscript before submission. All authors contributed to the article and approved the submitted version.

## Funding

ZT is supported by a Australian Government Research Training Program Scholarship.

## Acknowledgments

We thank BioRender.com for allowing us to create the figures throughout this review.

## Conflict of interest

The authors declare that the research was conducted in the absence of any commercial or financial relationships that could be construed as a potential conflict of interest.

## Publisher’s note

All claims expressed in this article are solely those of the authors and do not necessarily represent those of their affiliated organizations, or those of the publisher, the editors and the reviewers. Any product that may be evaluated in this article, or claim that may be made by its manufacturer, is not guaranteed or endorsed by the publisher.

## Glossary

**Table:**

BBB	Blood Brain Barrier
CNS	Central Nervous System
CSF	Cerebrospinal Fluid
cGAS	cGAMP Synthase
DNA	Deoxyribonucleic Acid
EGFR	Epidermal Growth Factor Receptor
HSV-1	Herpes Simplex Virus-1
IRF3	IFN Regulatory Factor 3
VSIV	Indiana Vesiculovirus
IRS1	Insulin Receptor Substrate 1
IFN	Interferon
ISGs	Interferon Stimulated Genes
JAK	Janus Kinase
JEV	Japanese Encephalitis
LGP2	Laboratory of Genetics and Physiology 2
LCMV	Lymphocytic Choriomeningitis Virus
MeV	Measles Virus
MDA5	Melanoma Differentiation-Associated Gene 5
MAVS	Mitochondrial Antiviral-Signaling Protein
MAPKs	Mitogen-Activated Protein Kinase
MyD88	Myeloid Differentiation Primary Response Gene 88
NF-κB	Nuclear Factor κ-Light-Chain-Enhancer of Activated B Cells
PAMPs	Pathogen-Associated Molecular Patterns
PRRs	Pattern Recognition Receptors
RABV	Rabies Virus
RIG-I	Retinoic Acid-Inducible Gene 1
RLRs	Retinoic-Acid-Inducible Gene I (RIG-I)-Like Receptors
RNA	Ribonucleic Acid
SINV	Sindbis Virus
STING	Stimulator of IFN Genes
TBK1	TANK-Binding Kinase 1
TMEV	Theiler’s Encephalomyelitis Virus
TBEV	Tick-Borne Encephalitis Virus
TRIF	TIR-Domain-Containing Adapter-Inducing Interferon-β
TIRAP/MAL	Toll/Interleukin-1 Receptor Domain Containing Adaptor Protein
TLR	Toll-Like Receptor
TRAM	TRIF- Related Adaptor Molecule
TRAF	Tumor-Necrosis Factor (TNF) Receptor Associated Factor
TYK	Tyrosine Kinase
WNV	West Nile Virus
ZIKV	Zika Virus
